# Editorial: Polycystic ovarian syndrome in adolescence

**DOI:** 10.3389/fendo.2026.1857004

**Published:** 2026-05-11

**Authors:** Rajni Sharma

**Affiliations:** Department of Pediatrics, All India Institute of Medical Sciences, New Delhi, India

**Keywords:** consensus guidelines, hyperandrogenism, hyperinsulinemia, menstrual irregularity, Rotterdam criteria

## Introduction

Polycystic ovarian syndrome (PCOS) is a common disorder of ovarian dysfunction affecting approximately 5-18% of adult female population (1). It is characterized by irregular menstrual cycles, hyperandrogenism and polycystic ovarian morphology (PCOM). PCOS is associated with several long-term complications including infertility, pregnancy-related complications, metabolic diseases (type 2 diabetes mellitus, hypertension, cardiovascular morbidity and mortality), endometrial cancer and mental health problems (2). Globally, PCOS is emerging as a major public health issue with rising healthcare expenditure related to its long-term chronic complications (3). The most commonly used criteria for the diagnosis of PCOS include the ESHRE-ASRM Rotterdam 2003 criteria which require the presence of two out of three features (oligomenorrhea, hyperandrogenism and PCOM on transvaginal ultrasound) (4).

In majority of women with PCOS, the symptom onset can generally be traced back to adolescence. However, diagnosis during the adolescent period is challenging. Irregular menstrual cycles may represent a normal physiological feature of pubertal transition, leading to underdiagnosis and missed opportunities for preventive and therapeutic intervention. Conversely, polycystic ovarian appearance of ovaries is relatively common in adolescence leading to overdiagnosis of PCOS. To address these challenges, the international consensus group published guidelines for the diagnosis of PCOS in adolescence. According to these recommendations, both menstrual irregularity and hyperandrogenism (clinical and/or biochemical) are mandatory for the diagnosis of PCOS in this age group (5). Adolescents presenting only with one (such as irregular menstrual cycles and isolated hirsutism) do not meet the full diagnostic criteria and are classified as “at risk for PCOS”. Such adolescents require follow up till 8 years post menarche for confirmation of PCOM to establish a definitive diagnosis (5, 6).

## Aims and objectives

There is limited research on the natural history and long-term outcomes of adolescent PCOS and those with “at risk” of PCOS diagnosed by the latest consensus guidelines. Emerging studies do suggest that the consensus guidelines help identify girls with PCOS with the highest risk for high BMI-related long-term complications (7, 8). The etiopathogenesis of PCOS is complex and linked related to genetic, environmental and lifestyle factors with hyperinsulinemia being central driving mechanism resulting in neuroendocrine and ovarian dysfunction. However, the role of hyperandrogenaemia in perpetuating obesity and hyperinsulinemia remains unclear and could be described as a ‘chicken and egg’ scenario and the exact pathogenetic mechanisms of PCOS remain to be fully elucidated (9). The current Research Topic aims to explore into the diagnostic features and pathophysiology of adolescent PCOS in greater depth and analyse its long-term implications.

## Overview of contributions

One study (Ozer et al.) examines the role of biochemical hyperandrogenaemia in the diagnosis of adolescent PCOS focussing on the diagnostic utility of serum free androgen index (FAI) and sex hormone binding globulin (SHBG). The authors suggest that FAI and SHBG may not effectively distinguish obese hyperinsulinemic girls from those with PCOS.

Another study (Wang et al.) reports the global trends in prevalence and incidence of PCOS among adolescents and young adults (10–24 years of age) from over 200 countries over the last 30 years and likely projection till 2036. The findings indicate an alarming rise in PCOS prevalence closely linked to the socioeconomic development as well as lifestyle and dietary factors. It is to note that the prevalence estimates of PCOS vary depending on the diagnostic criteria used for diagnosis and are lower when applying the updated consensus compared to Rotterdam criteria (10).

Obesity plays an important role in the etiopathogenesis of PCOS and the contribution of adipose tissue and its’ metabolic secretions continues to be investigated. One study (Orszulak et al.) explores the role of various adipokines in the pathophysiology of adolescent PCOS Another study (Skrzyńska et al.) evaluates the long-term implications of PCOS and the utility of endothelial dysfunction markers visfatin and vascular endothelial growth factor (VEGF) and their association with metabolic parameters in adolescent girls.

Additionally, one study (Prosperi and Chiarelli) provides a comprehensive overview of the complex bidirectional link between insulin resistance, metabolic complications and PCOS. Lastly, a detailed case report (Luo et al.) highlights the favourable response to time restricted feeding (intermittent fasting) in adolescent twins with PCOS. Collectively, these contributions enhance our understanding on the diagnostic challenges and mechanisms of adolescent PCOS.

## Future directions and call for action

There is a pressing need for longitudinal studies on the natural history of adolescents diagnosed with PCOS and “at risk” of PCOS to identify metabolic, reproductive and quality of life-related outcomes. Such studies would help identify clinical phenotypes with greatest risk of adverse outcomes. Furthermore, future studies should focus on identifying the specific genetic, environmental and individual predisposing factors in the development of PCOS with the aim to design effective preventive strategies and optimising therapeutic approaches. Currently, there are no standardised medication guidelines for adolescent PCOS and choice of therapy is highly individualised depending on patient needs. Although lifestyle modification is the cornerstone of management, adherence is often hindered by psychological and behavioural factors unique to adolescence. Additional research is required to establish optimal management protocols for adolescent PCOS in order to improve quality of life and reduce future complications.

## References

[1] Joham AE Norman RJ Stener-Victorin E Legro RS Franks S Moran LJ . Polycystic ovary syndrome. Lancet Diabetes Endocrinol. (2022) 10:668–80. doi: 10.1055/s-0041-1735506. PMID: 35934017

[2] Anagnostis P Tarlatzis BC Kauffman RP . Polycystic ovarian syndrome (PCOS): long-term metabolic consequences. Metabolism. (2018) 86:33–43. doi: 10.1016/j.metabol.2017.09.016. PMID: 29024702

[3] Azziz R Marin C Hoq L Badamgarav E Song P . Health care-related economic burden of the polycystic ovary syndrome during the reproductive life span. J Clin Endocrinol Metab. (2005) 90:4650–8. doi: 10.1210/jc.2005-0628. PMID: 15944216

[4] Group REA-SPCW . Revised 2003 consensus on diagnostic criteria and long-term health risks related to polycystic ovary syndrome (PCOS). Hum Reprod. (2004) 19:41–7. doi: 10.1093/humrep/deh098. PMID: 14688154

[5] Teede HJ Tay CT Laven JJE Dokras A Moran LJ Piltonen TT . Recommendations from the 2023 international evidence-based guideline for the assessment and management of polycystic ovary syndrome. J Clin Endocrinol Metab. (2023) 108:2447–69. doi: 10.2139/ssrn.4689131 PMC10505534 37580314

[6] Peña AS Witchel SF Boivin J Burgert TS Ee C Hoeger KM . International evidence-based recommendations for polycystic ovary syndrome in adolescents. BMC Medicine. (2025) 23(1):151. 40069730 10.1186/s12916-025-03901-w PMC11899933

[7] Tay CT Hart RJ Hickey M Moran LJ Earnest A Doherty DA . Updated adolescent diagnostic criteria for polycystic ovary syndrome: impact on prevalence and longitudinal body mass index trajectories from birth to adulthood. BMC Med. (2020) 18:389. doi: 10.1186/s12916-020-01861-x. PMID: 33302955 PMC7731536

[8] Kim JJ Hwang KR Lee D Kim S Choi YM . Adolescents diagnosed with polycystic ovary syndrome under the Rotterdam criteria but not meeting the diagnosis under the updated guideline. Hum Reprod. (2024) 39:1072–7. doi: 10.1093/humrep/deae042. PMID: 38514450

[9] Armanini D Boscaro M Bordin L Sabbadin C . Controversies in the pathogenesis, diagnosis and treatment of PCOS: focus on insulin resistance, inflammation, and hyperandrogenism. Int J Mol Sci. (2022) 23:4110. doi: 10.3390/ijms23084110. PMID: 35456928 PMC9030414

[10] Neven AC Forslund M Ranashinha S Mousa A Tay CT Peña A . Prevalence and accurate diagnosis of polycystic ovary syndrome in adolescents across world regions: a systematic review and meta-analysis. European J Endocrinol. (2024) 191(4):S15–27. 10.1093/ejendo/lvae125 39353075

